# Repatriation of Patients to Referral Centers and Outcomes After Kidney Transplantation: A Single-center Analysis From the United Kingdom

**DOI:** 10.1097/TXD.0000000000000834

**Published:** 2018-10-17

**Authors:** Khalid Khalil, James Hodson, Benjamin Anderson, Jay Nath, Adnan Sharif

**Affiliations:** 1 Institute of Immunology and Immunotherapy, University of Birmingham, Birminghamn, United Kingdom.; 2 Institute of Translational Medicine, Queen Elizabeth Hospital, Edgbaston, Birmingham, United Kingdom.; 3 Department of Nephrology and Transplantation, Queen Elizabeth Hospital, Edgbaston, Birmingham, United Kingdom.

## Abstract

**Background:**

The aim of this study was to compare posttransplant outcomes of kidney allograft recipients between those followed up at transplant centers and those that were repatriated back to referral renal units.

**Methods:**

We analyzed data for 1375 consecutive patients transplanted in a single center in the United Kingdom. Patients were stratified into 3 groups: (1) externally referred patients with repatriation back for external follow-up (repatriated, n = 463), (2) externally referred patients not repatriated and followed-up internally at transplant center (nonrepatriated, n = 365), and (3) internally referred patients within transplant center with continued internal transplant center follow-up (internal, n = 547). Patient and death-censored graft survival were compared between groups on both univariable and multivariable analyses.

**Results:**

Baseline comparisons found that the nonrepatriated group had increased risk for prolonged length of stay and delayed graft function compared with repatriated patients. The nonrepatriated group had significantly shorter survival compared to the repatriated patients (90.2% vs 94.1% at 5 years, *P* = 0.013), which persisted after adjustment for confounders on multivariable analysis (hazard ratio, 1.86; 95% confidence interval, 1.06-3.28; *P* = 0.032). Death-censored graft survival was not found to differ significantly between the 3 groups (*P* = 0.192).

**Conclusions:**

Our results provide reassurance regarding repatriation of care after kidney transplantation for the United Kingdom. Nonrepatriated patients are identified as a high-risk group for increased mortality, but further investigation is warranted to probe this heterogeneous group and validate in a non-United Kingdom cohort.

Kidney transplantation is the preferred modality of renal replacement therapy for patients with end-stage kidney failure, and confers significant advantages compared to dialysis with regards to mortality, quality of life and cost effectiveness for the majority of individuals.^1^ Careful posttransplant management is key to ensure long-term outcomes are optimal, and clinical practice guidelines have been published to aid those efforts (eg, from the UK Renal Association^2^ and Kidney Disease: Improving Global Outcomes [KDIGO]^3^). However, published guidelines all fail to address an important logistical question; is posttransplant care for patients superior if undertaken at the transplant center compared to repatriation back to referring nontransplant centers?

This has important resource implications for the long-term surveillance and management of kidney allograft recipients. For example, in the United Kingdom, there are 72 renal units (comprised from a total of 283 teaching hospitals, district general hospitals and/or satellite dialysis units distributed nationally), of which 23 are classed as transplantation centers where kidney transplantation surgery is undertaken (UK Renal Registry data available to view at; https://units.renal.org/index.pl). Each transplant center therefore undertakes kidney transplantation surgery for both internally referred patients and also externally referred patients from renal units within their immediate geographic region. It is unclear how many kidney allograft recipients are subsequently repatriated back to referring centers. Historical data from the United States suggest that 65% of outpatient clinic visits between 4 and 12 months are at the transplant center, dropping to approximately half beyond 12 months.^4^ However, there are no data with regard to difference in outcomes between these patients.

To the best of our knowledge, no study has been undertaken to investigate whether outcomes differ between patients followed up at transplant centers versus those repatriated back to referring renal units. We believe this is an important issue to investigate to inform healthcare providers, transplant professionals and patients with regard to optimal postkidney transplantation care. Therefore, the aim of this analysis was to investigate any difference in posttransplant outcomes for kidney allograft recipients stratified by follow-up center in the United Kingdom.

## MATERIALS AND METHODS

### Study Population

We undertook a retrospective cohort analysis of all consecutive kidney-alone transplants performed at a single center (University Hospital Birmingham [UHB]) in the United Kingdom between January 2007 and January 2017. Recipients of multiple organs, and those younger than 18 years were excluded. Data were electronically extracted by the Department of Health Informatics for every study recruit, with manual data linkage to additional electronic patient records. Patient and graft survival outcomes, delayed graft function rates, and rejection data were acquired and linked from NHS Blood and Transplant.

### Immunosuppression Protocol

All patients received the same immunosuppression over the study period, with minimization of tacrolimus exposure, in line with the SYMPHONY protocol.^5^ Induction therapy was with basiliximab (20 mg, ×2) and methylprednisolone (500 mg). Maintenance therapy included tacrolimus (target 12-hour trough level, 5-8 ng/L), mycophenolate mofetil (2 g daily with tapering to 1 g daily after 6 months) and maintenance corticosteroids. Biopsies were indication-based in the context of transplant dysfunction (categorized as ≥20% creatinine rise or new-onset proteinuria). Biopsy data were classified in accordance to latest Banff criteria.^6^

Long-term management protocols used at UHB were as follows. Episodes of acute cellular rejection were treated with a bolus of corticosteroids, with T-cell depletion therapy for steroid-resistant rejection. Antibody-mediated rejection was treated with antibody removal by plasmapheresis +/− IVIG. Viral serology (eg, polyoma virus) and/or anti-HLA antibodies were checked by indication basis, based on transplant dysfunction. Management protocols used at non-UHB referral units may have differed as clinical care is devolved to individual units.

### Definitions of Variables

The primary variable being analyzed was based on the location of patient follow up in the pretransplant and posttransplant periods. For the pretransplant period, the referring center was recorded in the electronic patient record, and categorized as internal (UHB) or external (a local renal unit). For the posttransplant period, the latest clinic letter for each patient was reviewed, to identify the center at which the most recent follow-up was performed (ie, internal or external). It was not possible to assess the timing of the transfer of posttransplant follow-up between centers, nor was it possible to identify those patients who may have been followed up externally, before returning to internal follow up due to complications. The cohort was then divided into 3 groups:

1. Repatriated: defined as end-stage kidney failure patients referred from a different center to UHB for kidney transplantation and subsequently repatriated back to the referring center for long-term follow-up.
2. Nonrepatriated: defined as end-stage kidney failure patients referred to UHB from a different center for kidney transplantation who remained at UHB for long-term follow up, rather than being repatriated to the referring local center.
3. Internal: defined as end-stage kidney failure patients who were being treated at UHB prior to kidney transplantation, and remained at UHB for long-term follow up.

For the externally referred patients, the protocol at the transplant center was for repatriation of all patients back to referring units (defined as repatriated group) regardless of preexisting risk status or referring center. Reasons for nonrepatriation (nonrepatriated group) are heterogenous and include; patient choice, clinical decision (eg, surgical complications, life threatening events, medical complexity), and logistical issues. However, these reasons were not recorded on an individual patient basis, and so could not be considered in the analysis.

Baseline and posttransplant data were extracted and classified from our database as follows. HLA mismatch levels were defined and graded in accordance to NHS Blood and Transplant classification (level 1: 000, level 2: 100, 010, 110, 200, 210, level 3: 020, 120, 220, 001, 101, 201, 011, 111, 211, and level 4: 021, 121, 221, 002, 102, 202, 012, 112, 212, 022, 122, 222). Matchability was calculated from a standardized pool of 10 000 recent donors, from which the numbers of blood group identical donors that recipients are well or favorably HLA-mismatched were counted. This number was converted to a standardized score between 1 and 10, which was used to categorize recipients into 1 of 3 matchability groups; easy,^1-3^ moderate,^4-6^ or difficult^7-10^ to match. Determination of socioeconomic deprivation was based upon the Index of Multiple Deprivation (IMD), a multiple deprivation model calculated at the local level area. The model is a composite construct of multiple domains reflective of area socioeconomic deprivation including: (1) income deprivation, (2) employment deprivation, 3) health deprivation and disability, (4) education skills and training deprivation, (5) barriers to housing and services, (6) living environment deprivation, and (7) crime. Individual domains are measured in isolation and subsequently combined (using appropriately judged weighting) into a single-composite value. The quintiles of the national distribution of IMD values were then combined to form 3 groups: more deprived (first and second quintile), intermediate (third quintile), and less deprived (fourth and fifth quintiles).

Data for patient and graft survival outcomes were acquired from NHS Blood & Transplant, and cross-referenced against internal databases to maximize data completeness. Follow-up commenced at the time of transplant, with patients censored at the earliest of: their final recorded follow up, the end of follow up for the study (September 1, 2017) or 5 years after transplant. For analysis of graft survival, patients that died with a working graft were censored at death.

### Statistical Analysis

Initially, a range of demographic and transplant characteristics were compared between the 3 follow up groups. Comparisons of ordinal and continuous variables were performed using Kruskal-Wallis tests, with χ^2^ tests used for nominal variables. Survival outcomes were then assessed with Kaplan-Meier curves and log-rank tests, with univariable Cox regression models used to produce hazard ratios (HRs). For the survival analyses, continuous variables were divided into groups either based on established cutoff values, or by using the percentiles of the distribution to give groups of similar sample size.

To account for the impact of potentially confounding factors, a multivariable analysis was then performed. Multivariable Cox regression models were produced, using a forward stepwise approach to select significant independent predictors of patient outcome. To minimize exclusions due to missing data, any factors that had data unavailable in greater than 10% of the cases and were nonsignificant in either the univariable or multivariable models were excluded from consideration, and the analysis was repeated. The factors in the final parsimonious model were then entered into a new model alongside the follow up group, in cases where this factor was not selected by the stepwise procedure.

All analyses were performed using IBM SPSS 22 (IBM Corp. Armonk, NY), with *P* less than 0.05 deemed to be indicative of statistical significance throughout.

### Approvals

This study received institutional approval and was registered as an audit (audit identifier; CARMS-12578). The corresponding author had full access to all data.

## RESULTS

### Study Cohort

Between January 2007 and January 2017, data were recorded for a total of 1393 consecutive kidney-alone transplants at the transplant center (UHB). Of these, there was a small number of cases (N = 18) that were treated at UHB pretransplant, but followed up externally posttransplant who were excluded, due to insufficient sample size in this subgroup. For the remaining 1375 transplants, 828 were referred to UHB for transplant from 1 of 6 local centers, of whom 463 (55.9%) were repatriated after transplant, and 365 (44.1%) had posttransplant follow up at UHB (nonrepatriated). The remaining 547 were internal patients that were followed up at UHB both pretransplant and posttransplant.

### Baseline Comparisons

Tables 1 and 2 highlight multiple differences in the baseline characteristics and transplant profiles between the 3 patient groups. Significant differences in distributions of both gender (*P* = 0.004) and ethnicity (*P* < 0.001) were detected, with the repatriated patients having a higher proportion of males and white patients than the other 2 groups. Internally referred patients were the most likely to have received previous transplants at 13.6%, compared with 7.0% and 6.4% in repatriated and nonrepatriated external referrals, respectively (*P* < 0.001). Rates of ABO-incompatible transplantation also differed across the groups (*P* = 0.006), with only 3.0% of repatriated patients receiving such organs, compared with 7.7% of nonrepatriated and 6.9% of internal patients. Cold ischemia times (CITs) were longest in repatriated patients, at a median of 840 minutes, compared to 749 minutes in nonrepatriated patients, and 715 minutes in internal referrals (*P* = 0.048).

**TABLE 1:**
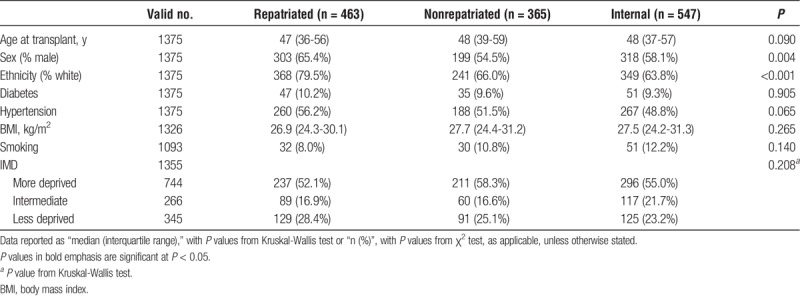
Baseline demographics of the study population

**TABLE 2:**
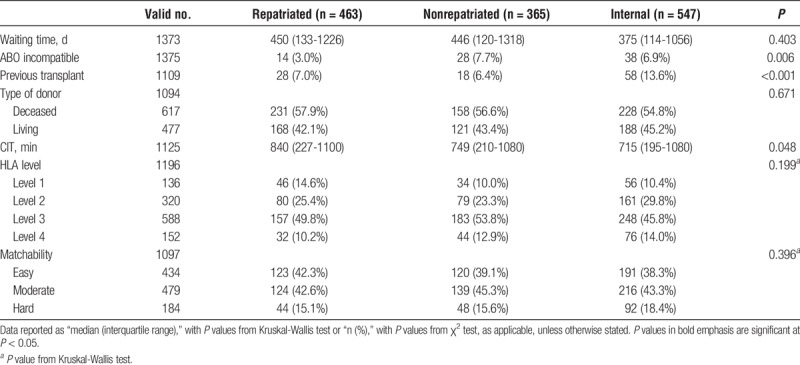
Transplant characteristics of the study population

### Posttransplant Complications

Of the short-term outcomes considered, significant differences between the groups were detected in postoperative length of stay (*P* = 0.008) and delayed graft function (*P* = 0.016) (see Table 3). In both cases, it was the repatriated group that had the better outcomes, with a shorter length of stay (median, 8 days vs 9 days in other groups) and a lower rate of delayed graft function (8.5% vs 12.6% and 15.6% in the nonrepatriated and internal groups, respectively). A significant difference in the rate of biopsy-proven BK neuropathy was also detected between the groups, which was again lower in the repatriated patients (2.0% vs 4.6% and 5.5% in the nonrepatriated and internal groups, respectively, *P* = 0.033). No significant differences were detected between the groups in the rates of de novo HLA antibody (*P* = 0.102) or either biopsy proven rejection (*P* = 0.410) or disease recurrence (*P* = 0.544) in the posttransplant period.

**TABLE 3:**
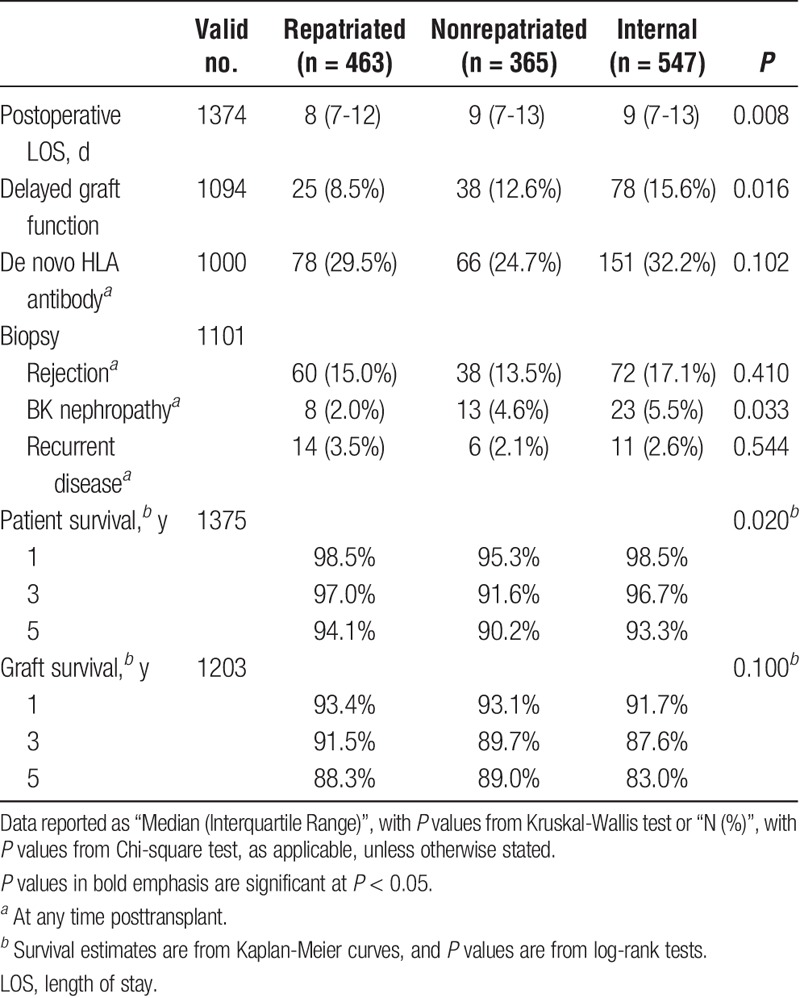
Posttransplantation outcomes of the study population

### Patient and Graft Survival by Follow-up Group

**FIGURE 1:**
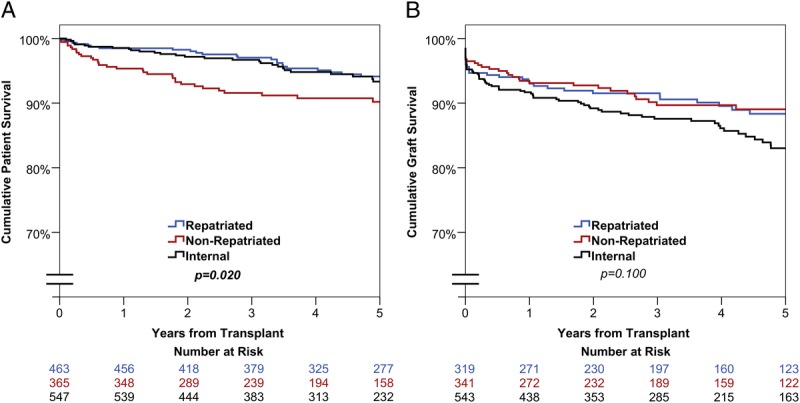
Kaplan-Meier curves of (A) patient and (B) graft survival by patient group.

The median patient follow up was 58 months (interquartile range, 34-60), during which time there were 83 deaths. Graft follow-up was only recorded in 1203 cases, with a total of 137 graft losses occurring during the study period. Kaplan-Meier curves comparing both outcomes between transplant groups are shown in Figure 1. Patient survival was found to differ significantly between the 3 groups (*P* = 0.020). Of the externally referred patients, those that were repatriated had the longest survival, with 94.1% of patients surviving for at least 5 years posttransplant compared with 90.2% of nonrepatriated patients (HR, 1.97; 95% CI, 1.15-3.37; *P* = 0.013). Internally referred patients had similar survival to those that were repatriated, with rates of 94.1% versus 93.3% at 5 years (HR, 1.12; 95% CI, 0.65-1.95; *P* = 0.686). No significant difference in death-censored graft survival was detected between the 3 patient groups (*P* = 0.100).

### Other Predictors of Survival

In addition to the observed differences between the 3 follow-up groups, patient survival was found to be significantly shorter with increasing age at transplant (*P* < 0.001), in longer postoperative lengths of stay (*P* < 0.001) and in recipients of deceased donor organs (*P* = 0.021, Table 4). Analysis of graft survival found this to be shorter in patients that were smokers (*P* = 0.012), of nonwhite ethnicity (*P* = 0.027), had received previous transplants (*P* = 0.009) or a deceased donor organ (*P* < 0.001), and in those with longer CIT (*P* < 0.001), more difficult matchability (*P* = 0.005), longer postoperative lengths of stay (*P* < 0.001), or delayed graft function (*P* = 0.002).

**TABLE 4:**
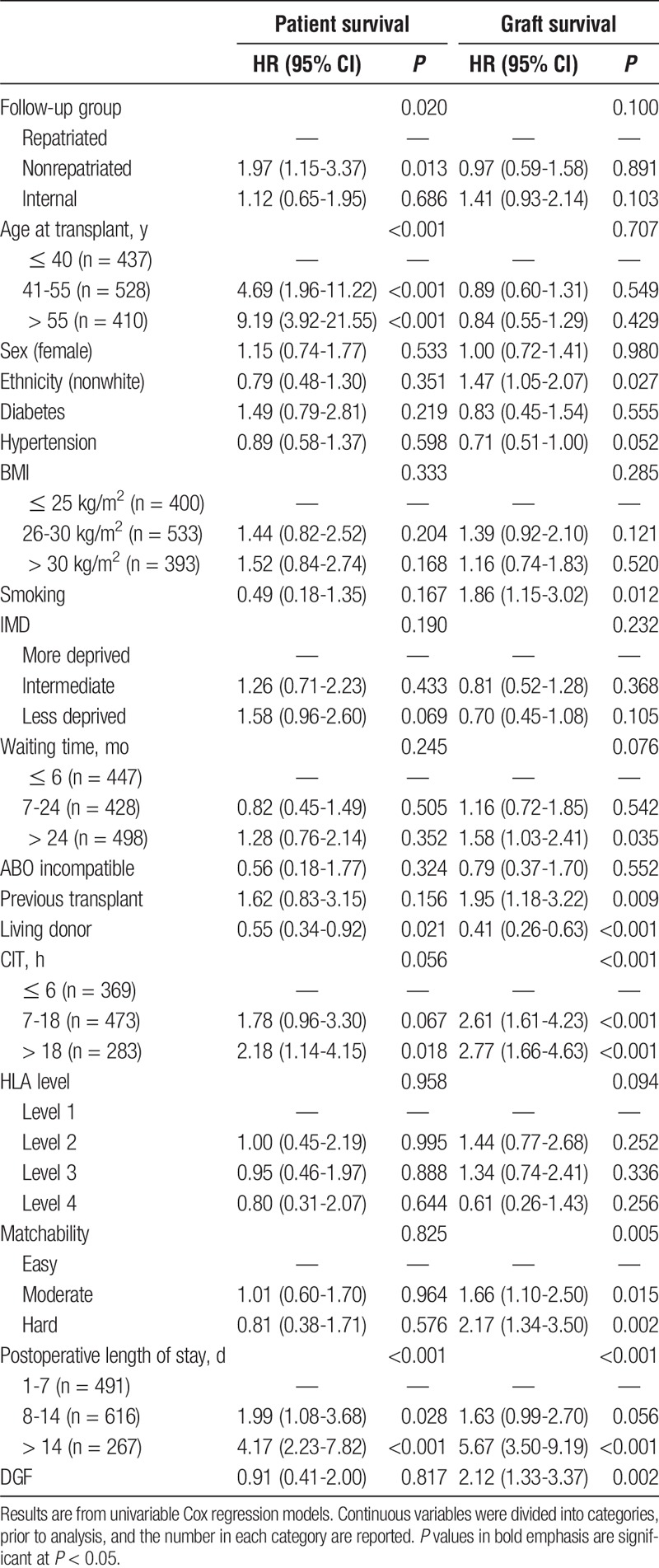
Univariable analyses of factors predictive of patient and graft survival

To account for impact of these potentially confounding factors on the comparisons between the follow up groups, a set of multivariable analyses were performed (Table 5). Analysis of patient survival found increasing patient age at transplant (*P* < 0.001), postoperative length of stay (*P* = 0.004) and previous transplantation (*P* = 0.014) to be independently predictive of shorter patient survival. After accounting for these factors, the difference between the patient follow up groups remained significant (*P* = 0.037), with the nonrepatriated patients having significantly poorer overall survival than those that were repatriated (HR, 1.86; 95% CI, 1.06-3.28; *P* = 0.032). Patient survival was almost identical in the repatriated versus internally followed up groups, with an HR of 1.00 (95% CI, 0.55-1.80; *P* = 0.992). Analysis of graft survival found only the postoperative length of stay to be independently predictive of this outcome (*P* < 0.001). After accounting for this factor, no significant difference was detected between the patient follow-up groups (*P* = 0.192).

**TABLE 5:**
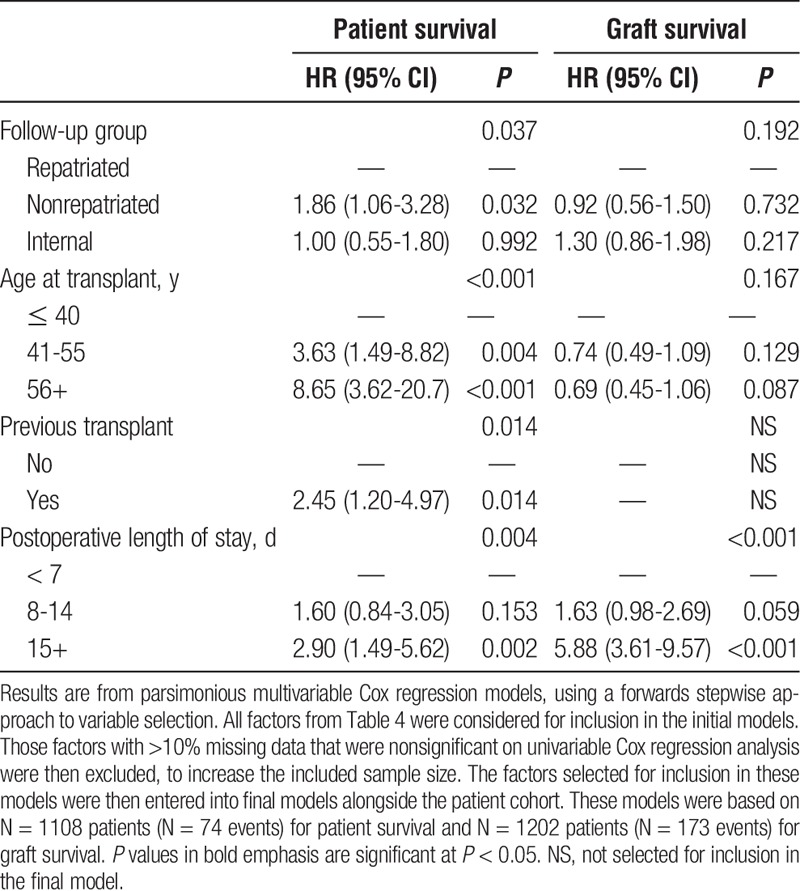
Multivariable analyses of factors predictive of patient and graft survival

## DISCUSSION

To the best of our knowledge, there are no data in the literature to compare postkidney transplant outcomes between patients followed up at transplant centers that undertake transplantation surgery versus those repatriated back to referring renal units that do not perform transplantation surgery. In this retrospective single-center analysis in the United Kingdom, we have highlighted worse patient survival in the nonrepatriated cohort, which persisted on multivariable analysis after adjustment for competing factors. No significant difference in patient or graft survival was observed between repatriated and internal patients. Our results provide reassurance for healthcare providers, transplant professionals, and patients in the current practice of repatriating kidney allograft recipients posttransplantation and identify a unique cohort of patients who should be considered at higher risk for posttransplant mortality. However, they represent a heterogeneous group specific to the United Kingdom. Therefore, further work is required to probe the nonrepatriated cohort and validation in an international cohort is warranted.

When interpreting our results, it would be oversimplistic to treat patients as being under either specialist or generalist transplant care, as continued professional engagement is likely, even when kidney transplant recipients are repatriated from transplant centers. Shared decision making and consultation between colleagues may lead to bidirectional transfer of recipients when required for specialist services as warranted. While specialist clinicians may be more up-to-date with transplantation research, there is conflicting evidence in the literature that conference attendance leads to improved performance. For example, no correlation is observed with conference attendance and in-training examination results in internal medicine^7^ or emergency medicine,^8^ but improvements have been observed in surgical board examination results.^9^ No data are available for transplantation conference attendance, and this would be an interesting area for future analysis.

Finally, it is unclear whether managing a larger cohort of transplant patients (eg, transplant centers vs nontransplant centers) leads to improved care—the so-called volume-outcome relationship.^10^ Research has shown increased dialysis center volume leads to improved peritoneal dialysis outcomes and possible advantage on hemodialysis outcomes.^11,12^ This would fit in with general consensus that increased center volume is positively associated with improved healthcare outcomes across a wide range of procedures and conditions of varying magnitude.^13^ However, the reciprocal has also been observed among hemodialysis patients, with increased mortality observed among patients receiving treatment from higher-caseload versus lower-caseload nephrologists^14^ and multiple competing and confounding factors will cloud any clear cause-and-effect association. Data relating to transplant center volume and outcomes are limited, with some data from the United States linking kidney transplant center performance (based on observed/expected events and standardized mortality ratios), rather than volume, to improved survival benefit postkidney transplantation.^15^ Although a replication of such analyses, separating follow up between transplant center and referring renal unit data, would be useful to investigate a center-specific performance effect on posttransplant outcome, registry data in the United Kingdom only report outcomes by transplant center. More granular data analysis would require significant changes to the upload of data to national registries, but should be explored for more detailed investigations.

The justification for this study was the perception that postkidney transplantation outcomes may be different between transplant centers (with care from specialist transplant nephrologists) and nontransplant centers (with care from more generalist nephrologists). There has been much discussion in the medical and surgical literature with regard to comparison of outcomes for specialist versus generalist care in the context of certain medical or surgical admissions. For example, Smetana and colleague undertook a systematic review of studies comparing specialist versus generalist care for individual patients with a single discrete medical condition.^16^ Of 49 included studies they observed the following; 24 studies favored specialist care, 13 studies found no difference in outcomes, 7 studies varied by individual outcome, 1 study was dependent on physician experience, and 4 studies favored generalist care. Numerous methodological shortcomings were identified across these heterogeneous studies including selection bias, small numbers and varied analytical techniques. How relevant these data, relating to admissions for single medical conditions, are in the setting of kidney transplantation is questionable, as kidney allograft recipients often comprise multiple chronic medical conditions and comparative effectiveness research in the setting of multiple chronic health issues is limited.^17^ However, our data do not support any notion that patient outcomes at generalist nephrology centers are poorer than specialist transplant centers.

The limitations of our epidemiological analysis should be appreciated in the interpretation of our data. The timing of patient repatriation differed across referral renal units, with some preferring early repatriation (upon discharge from inpatient transplant surgery episode) compared to others preferring late repatriation between 3 and 6 months postoperatively. This time-dependent confounder was not easily ascertainable from the electronic patient records and therefore was not factored into our analysis. As previously mentioned, there can be significant fluidity in patient flow, with bidirectional transfers between transplant centers and referral renal units to confound the data. Due to small numbers, we also did not undertake any subgroup analyses to stratify results across referral units to determine any difference between them. Indeed, given the few outcomes (eg, deaths, graft loss) in the 3 strata, a multivariable analysis of retrospective data may not capture the full spectrum of risk factors due to insufficient statistical power. Individual practice environments are likely to differ between regional transplant centers and external validity of this data may be limited. Typical of epidemiological analyses of cohort studies such as these, there are likely to be confounders that have an impact on mortality and graft loss postkidney transplantation that we were unable to include in the analysis, due to data not being available (eg, lifestyle factors, hospitalization episodes/illnesses, etc.). Missing data (and misclassification bias) also have an implication on the analyses performed, which is an inherent bias in epidemiological analyses such as this. This study is specific for the United Kingdom and the findings may not be translatable to settings outside of the United Kingdom with different infrastructure, financial reimbursement and follow-up models. However, as the repatriation of patients after kidney transplantation is standard practice at most transplant centers, we believe these results should be of interest globally. As such, we feel that further validation in a non–United Kingdom setting is warranted. With an adequately sized cohort, the possibility of using propensity score techniques for statistical matching could be considered, but the significant overlap of groups and residual confounding from unmeasured variables will likely cloud such analysis.^18^ In addition, long-term outcomes will be required to ascertain any true difference, and the limitations of registry analyses, especially for long-term survival analyses,^19^ highlight the importance of more collaborative research with record linkage to maximize transplant epidemiology research.^20^

To conclude, our study is the first to describe outcomes after kidney transplantation for patients stratified by the location of their posttransplantation care in the United Kingdom. Reassuringly, it shows equivalent patient and graft survival outcomes when comparing repatriated versus internally referred patients with continued internal transplant center follow-up. Patient survival was found to be significantly worse in externally referred nonrepatriated patients. Although further investigation on this complex and heterogeneous group is warranted, our results are reassuring, suggesting that outcomes for repatriated patients are no worse than those followed up internally at the transplant center.
